# Physico-Chemical Properties Prediction of Flame Seedless Grape Berries Using an Artificial Neural Network Model

**DOI:** 10.3390/foods11182766

**Published:** 2022-09-08

**Authors:** Adel M. Al-Saif, Mahmoud Abdel-Sattar, Abdulwahed M. Aboukarima, Dalia H. Eshra, Krzysztof Górnik

**Affiliations:** 1 Department of Plant Production, College of Food and Agriculture Sciences, King Saud University, P.O. Box 2460, Riyadh 11451, Saudi Arabia; 2 Pomology Department, Faculty of Agriculture, Alexandria University, El-Shatby, Alexandria 21545, Egypt; 3 Department of Agricultural Engineering, College of Food and Agriculture Sciences, King Saud University, P.O. Box 2460, Riyadh 11451, Saudi Arabia; 4 Agricultural Engineering Research Institute, Agricultural Research Center, Nadi El-Said St. Dokki, Giza 12619, Egypt; 5 Food Science and Technology Department, Faculty of Agriculture, Alexandria University, Alexandria 21545, Egypt; 6 The National Institute of Horticultural Research, Konstytucji 3 Maja 1/3, 96-100 Skierniewice, Poland

**Keywords:** agricultural practices, chemical properties, grape, quality, machine learning, modeling, physical properties

## Abstract

The grape is a very well-liked fruit that is valued for its distinct flavor and several health benefits, including antioxidants, anthocyanins, soluble sugars, minerals, phenolics, flavonoids, organic acids, and vitamins, which significantly improve the product’s overall quality. Today’s supply chain as a whole needs quick and easy methods for evaluating fruit quality. Thus, the objective of this study was to estimate the quality attributes of Flame Seedless grape berries cultivated under various agronomical management and other practices using color space coordinates (berry L*, berry a*, and berry b*) as inputs in an artificial neural network (ANN) model with the best topology of (3-20-11). Satisfactory predictions based on the R^2^ range, which was 0.9817 to 0.9983, were obtained for physical properties (i.e., berry weight, berry length, and berry diameter as well as berry adherence strength) and chemical properties (i.e., anthocyanin, total soluble solids (TSS), TSS/titratable acidity, total sugars, titratable acidity, reducing sugars, and non-reducing sugars). Meanwhile, we also performed a contribution analysis to analyze the relative importance of CIELab colorimeter parameters of berries L*, a*, and b* to determine the main fruit quality. In terms of relative contribution, berry b* contributed relatively largely to berry weight, berry adherence strength, TSS, TSS/titratable acidity, titratable acidity, total sugars, reducing sugars, and non-reducing sugars and a* contributed relatively largely to anthocyanin, berry length, and berry diameter. The developed ANN prediction model can aid growers in enhancing the quality of Flame Seedless grape berries by selecting suitable agronomical management and other practices to avoid potential quality issues that could affect consumers of them. This research demonstrated how color space coordinates and ANN model may well be utilized to evaluate the Flame seedless grape berries’ quality.

## 1. Introduction

Grape (*Vitis vinifera* L.) belongs to the family Vitaceae, which contains more than 60 genera and is native to the riverbanks of North America, Europe, and Asia [1,2]. It is an adaptable fruit shrub that grows in hot tropical, subtropical, and temperate climates in a variety of soil types [3,4]. The majority of the world’s grapes-growing regions, known as the temperate climatic belt, are found between the latitudes of 40° and 50° N in the Northern hemisphere and between latitudes of 30° and 40° S in the Southern hemisphere [3]. Grapevines occupy a lot of land in the world (7515 thousand ha), with a total production of 77.3 million tons, of which 36% corresponded to fresh table grapes in 2015 [5]. In Egypt, it is ranked second after citrus fruits, due to its high net return; it is grown rapidly, especially in reclaimed soils [6,7]. Due to the availability of antioxidants, minerals (such as calcium, magnesium, and iron), soluble sugars, anthocyanins, flavonoids, organic acids, vitamins (such as B1, B2, and C), and aromatic compounds, the grapevine is one of the most economically significant sources for the human health and food industries [8,9,10,11]. Furthermore, the grapevine has rich phenolic compounds, so it has positive health effects on people to protect them from different diseases [12,13,14,15,16]. Additionally, it is used in food additives, the pharmaceutical industry, and natural cosmetic products [17].

Flame Seedless is one of the most well-liked fruits in Egyptian markets because it is seedless and ripens the earliest [18,19]. It is one of the most widely cultivated table grapes in the world because it creates attractive clusters, and has a higher percentage of anthocyanin content than other seedless cultivars [20,21]. The marketing value of Flame Seedless grapes is based on their appealing fruit, cluster, and size/shape characteristics. Therefore, in order to assure profitability, it is essential to maximize yield and quality [20,22].

Grape quality and its nutrient composition vary depending on agronomical management practices (fertilization, irrigation, weed, and pest control), agrochemicals treatments (such as kaolin, hormones, and sucrose), viticultural (grape cultivars, cluster thinning, pruning, and trunk girdling), biotechnological techniques and growth stage, and environmental changes (soil, climate, and season) as well as combinations of leaf removal and crop load treatments [15,23]. Generally, fruit quality refers to a combination of traits that determine whether the fruit is suitable for consumption while still fresh or for storage for an appropriate amount of time without deterioration and providing advice on its worth in terms of customer acceptance [24,25]. Only high-quality fruit can increase a fruit’s ability to compete in the market. As people’s living standards have continued to rise in recent years, their expectations for nutritional content and fruit quality have also increased [26]. Most methods to assess the internal quality of fruits are based on laboratory measurements. However, these measurements are traditional methods requiring samples, which is time-consuming, costly, and labor-intensive [27]. Similar to external quality forecasts, numerous techniques have been created and applied; these techniques rely on mathematical models and statistical regressions. When compared to traditional sample-based measurements, image processing has the ability to quickly capture a fruit’s dimension and color. Additionally, machine learning methods, such as artificial neural networks (ANN), have been used to predict fruit quality [28]. The development of methods for evaluating the quality of fruits and vegetables has received significant attention recently, and tools of quality assessment have gained momentum [29]. Moreover, different studies examined ANN for determining the quality of different fruit and vegetables with the aid of some features that include fruit color, size, etc. [28,30]. Although determining the quality of any food material is a challenging task, ANN technologies and their intelligent capabilities make them both highly accurate and economically advantageous [29]. Additionally, ANN is capable of learning without human intervention. It can treat existing data internally through different activation functions, adjust weights for different settings, examine hidden associations between data, and, therefore, represent unknown data, which is of different significance for prediction. An ANN’s powerful computing power lets it treat more data and solve many identical multifaceted nonlinear problems [30]. As an example, Amoriello et al. [28] collected data for seven strawberry varieties and created two statistical procedures, i.e., multiple linear regression (MLR) and ANN models, to predict titratable acidity, dry matter, soluble solids, and firmness as well as nutritional attributes, such as antioxidant potential, total anthocyanins, and total phenols. The inputs to the models were color space coordinates, which were denoted by L*, a*, and b* of strawberries. When MLR was used, unsatisfactory prediction performances were achieved for all parameters. Contrarily, the quality attributes were accurately predicted by ANN, particularly for antioxidant activity and total monomeric anthocyanin, with higher coefficients of determination above 0.90. The study showed that color coordinates and the established ANN models could be successfully utilized to assess strawberry quality. Huang et al. [30] applied ANN to discover the effects of mineral nutrients in leaves and soil on the fruit quality attributes of loquat. The results indicated that the developed ANN models with different topologies could predict the titratable acid content, the single fruit weight, and soluble solids content with higher coefficients of determination. Sun et al. [31] predicted peach fruit quality of single fruit weight, fruit soluble solids content, and fruit titratable acid content using ANN based on soil mineral nutrients and showed high prediction accuracy based on R^2^ values over 0.90.

Optimizing the use of suitable management measures, particularly agronomical management and other practices, is necessary to prevent a harmful imbalance between vine growth and quality. Moreover, the quality attributes of Flame Seedless grape berries can be analyzed qualitatively using laboratory analysis. However, many previous research papers [28,30] have specified that the ANN technique is a very operative and consistent predicting tool, which has been extensively used in fruit quality field and has a very high prediction precision. The present research aimed to notice the dependability of the ANN model to predict the berry weight, berry length, and berry diameter as well as berry adherence strength and chemical properties (i.e., anthocyanin, total soluble solids (TSS), TSS/titratable acidity, total sugars, titratable acidity, reducing sugars, and non-reducing sugars) of Flame Seedless grape berries and to discover the effects of CIELab colorimeter parameters of berries L*, a*, and b* on the key fruit quality attributes of them. This was the aim so as to provide a theoretical source for quality attribute diagnosis and a boost in choosing the right agronomical management and other practices in practical production to avoid potential quality issues that could affect consumers of Flame Seedless grape berries.

## 2. Materials and Methods

### 2.1. Vineyard’s Description

The lack of readily available valuable data for training and validation purposes is the key obstacle to adopting machine learning procedures in the horticulture industry for the aim of predicting fruit quality [32]. Thus, the fresh grape samples for the required data were collected from seven-year-old Flame Seedless grapevines, which were cultivated in private vineyards located at QoraKhargin, Gharb El-Nobarya region, Beheira Governorate, Egypt (30°34′55.3″ N, 29°53′25.4″ E) during the 2021 growing season. The vines were planted on sandy soil with an average pH of 7.7–7.8 and were spaced 4 × 1.75 m apart. They were watered using a drip irrigation technique with two lines and four drippers (8 L/h) per vine. Nine vineyards that vary in agronomical management and other practices (such as hoeing, irrigation, fertilization, pinching, pest management, etc.) from one vineyard to another were chosen for collecting the required data in the present study. Additionally, 81 vines were chosen to be as uniform as possible in growth, with no visible signs of nutrient deficiency, and almost uniform in their vigor. The grape samples were collected from nine vineyards with nine replicates per vineyard and two vines per replicate.

### 2.2. Measurements of Studied Parameters

When the grape berries got their peak color in the second week of May 2021, the grape clusters were gathered and brought to the laboratory at the Faculty of Agriculture, Food Science and Technology Department, Alexandria University, Egypt, for sample analysis. The berries from each vine’s cluster were carefully separated manually, and fifteen berries were obtained from each vineyard for determination of berry weight, berry length, and berry diameter immediately as the samples were not stored. Berry weight was recorded using a digital scale. However, berry length and berry diameter were measured using a digital caliper. Furthermore, a digital force gauge (DPS-110R, Imada, Northbrook, IL, USA) was also used to measure the strength of the berry adhesion, which refers to separation force in g, which is then converted to N.

Another random sample of berries was collected to determine the chemical properties. In the juice, the percentage of total soluble solids (TSS) was measured by a hand refractometer (Atago, Tokyo, Japan). The titratable acidity expressed as grams of tartaric acid/100 mL of juice was determined according to AOAC [33], and the ratio between TSS and titratable acidity was calculated. Anthocyanin pigment in the berries’ skin (mg/100 g fresh weight) was determined using spectrophotometry at a wavelength of 535 nm, according to Ranganna [34]. Total sugars were determined by using the phenol sulfuric acid method outlined by Malik and Singh [35]. Reducing sugar content was determined according to the Lane and Eynon method as described by Egan et al. [36]. The difference between total sugars and reducing sugars was used to compute the non-reducing sugar levels.

Berries’ color was evaluated using a colorimeter (CR-3000, Konica Minolta, Tokyo, Japan), which measures L*, a*, and b* values. The L* value is equivalent to a dark–bright scale (0, black; 100, white), the a* value to a green–red scale (negative value, greenness; positive, redness), and the b* value to a blue–yellow scale (negative value, blueness; positive, yellowness).

### 2.3. Statistics Analysis

All data were analyzed for vineyard effects on measured quality attributes by ANOVA using SAS [37]. At a probability level of 0.05, the means were divided and compared using the least significant difference (LSD) method. Further, descriptive statistics, such as Kurtz, mean, maximum, skewness, minimum, and standard deviation, were achieved; however, coefficients of variation were calculated based on standard deviation and mean values to evaluate the dispersed of the collected data. The descriptive statistics were calculated using SPSS statistical software (version 22, SPSS, Chicago, IL, USA), where the dataset comprised of 81 observations. Additionally, the pair relationships among all of the quality attributes were assessed using the Person correlation coefficient.

### 2.4. Artificial Neural Network Modeling

**Figure 1:**
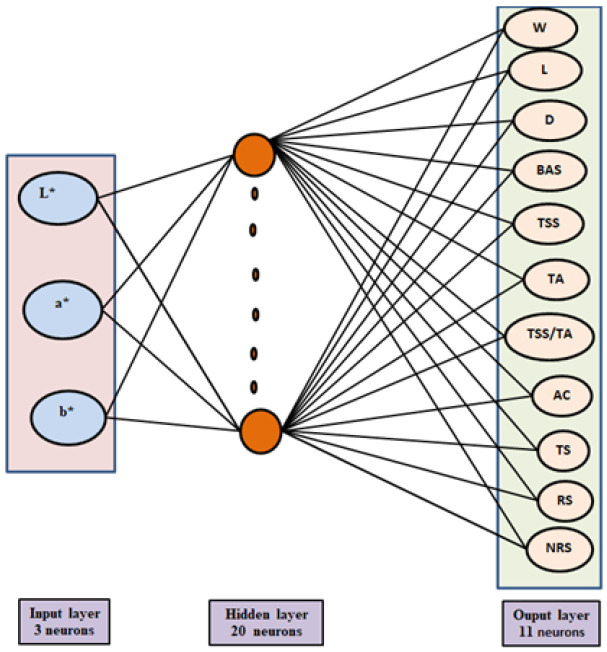
Structure of the multi-layer perceptron artificial neural network. The output parameters are berry weight (W), berry adherence strength (BAS), total soluble solids content (TSS), berry length (L), berry diameter (D), titratable acidity (TA), TSS/titratable acidity, anthocyanin (AC), total sugars (TS), reducing sugars (RS), and non-reducing surges (NRC). The input variables are L* = lightness; a* = redness; b* = yellowness.

The quality attributes of Flame Seedless grape berries cultivated under various agronomical management and other practices were predicted using an ANN model. The ANN was a nonlinear model constructed using a feed-forward architecture as the multi-layered perceptron, with back propagation as the training algorithm. It has one input layer, in which the neurons (L*, a*, and b*) operate as independent parameters, one or more hidden layers, and one output layer, in which each output parameter acts as a dependent parameter on the neurons (Figure 1). The output variables were berry weight, berry length, berry diameter, berry adherence strength, TSS, titratable acidity, TSS/titratable acidity, anthocyanin, total sugars, reducing sugars, and non-reducing sugars.

The standard back propagation algorithm was used, an iterative technique that trained the dataset by randomly dividing the complete collection of data into training (80% of the data) and testing sets (20% of the data). The network was trained using the training dataset to produce output values as close to the target values as possible, as the learning algorithm was used to update the weights, which are connected to the associates between the neurons. The network performance after the learning phase was evaluated using the test dataset. The neurons of the first layer take the input values from fed data during the training phase, each of which is weighted independently, and error minimization is achieved by the back propagation algorithm. The size of the input vector is equal to the number of artificial processing neurons or nodes. The activation function is applied after each input node sends a signal to each hidden node as a weighted sum. The signal from the hidden layer to the output layer was also subject to the same procedure. The hidden (*x_i_*) and output (*y_i_*) neuron activities are defined as follows [28]:

(1) $$x_{i}=f\left(v_{i}\right)$$

(2) $$y_{i}=f\left(v_{i}\right)$$

where the activation function used in the output or hidden layers is *f*(*v_i_*). Due to its improved performance, feed-forward neural network models typically use entire topologies with hidden layers and an active transfer function, which is the sigmoid in this study [38]. The activation function (sigmoid function) is as follows [28]:

(3) $$f\left(v_{i}\right)=v_{i}$$

(4) $$f(v_{i})=\frac{1}{1+e^{-v_{i}}\;}$$

**Figure 2:**
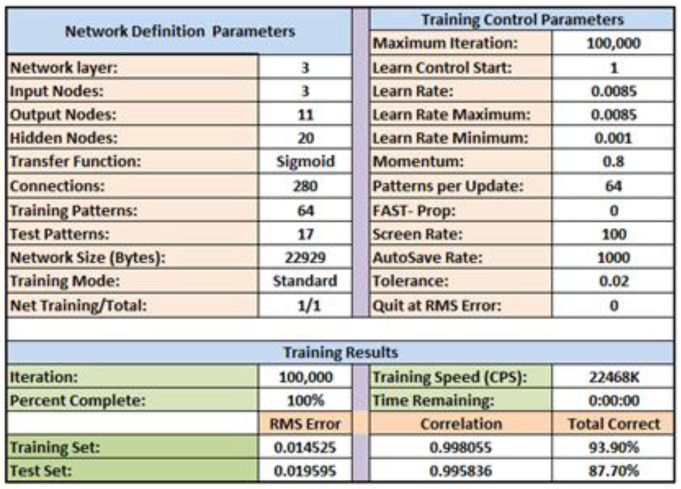
Network definition and training control parameters of the best topology of the ANN model (3-20-11) to predict quality attributes of Flame Seedless grape berries cultivated under various agronomical management and other practices acquired from Qnet2000 software.

*ν_i_* is calculated as follows:

(5) $$v_{i}=\sum_{j=1}^{m}w_{ij}\;x_{j}+b_{i}$$

where *m* is the total number of nodes in the output layer, *w_ij_* is the weight between layers *i-th* and *j-th*, and *b_i_* is the bias of the *i-th* neuron. The network in this study was trained 100,000 times with initial weight and bias values selected at random by the software, and the hidden layer was tested with a range of topologies and neurons from 1 to 30. To build the ANN model, we used a commercial neural network software called Qnet2000 on Windows, Vesta Services, 2000 [39]. However, the input and output values were normalized between 0.15 and 0.85 by the software, according to the following equation.

(6) $$T=\frac{\left(t-t_{\min}\right)}{\left(t_{\max}-t_{\min}\right)}\times (0.85-0.15)+0.15$$

where *t* symbolizes the actual values of the input and output variables, *T* is the normalized value, and *t*_min_ and *t*_max_ are the minimum and maximum values of input and output variables, respectively. However, Figure 2 shows the information of the best topology of the ANN model (3-20-11) with network definition and training control parameters to predict the quality attributes of Flame Seedless grape berries cultivated under various agronomical management and other practices. However, during the building of the ANN model employing a feed-forward type based on an error back propagation algorithm, the trial-and-error procedure was employed to get the optimal ANN configuration. Additionally, a major challenge in training neural networks is knowing when to stop training. If we train too little, the model will underfit the training and test sets. If we train too much, the model will overfit the training set and have poor performance on the test set. We noticed that the total corrected percentage (87.7%), as shown in Figure 2, of the testing dataset stops early if the performance starts to degrade on a validation dataset. However, a flow chart tagging the different solving steps for establishing the present ANN model using the applied software of Qnet2000 is explained in a previous study [40].

### 2.5. Evaluation of the Performance of ANN Models

The presentation of the ANN model was appraised by some statistical indexes, such as mean absolute error (*MAE*), and root mean square error (*RMSE*), as follows:

(7) $$RMSE=\sqrt{\frac{1}{n}\sum_{i=1}^{n}{\left(P_{i}-O_{i}\right)}^{2}}$$

(8) $$MAE=\frac{1}{n}\sum_{i=1}^{n}\left|P_{i}-O_{i}\right|$$

where *O_i_* and *P_i_* are the measured and predicted values, respectively, and *n* is the total number of observations in each dataset. Moreover, scatter plots are offered for pictorial correlation of the predicted and measured values. The average magnitude of error denoted by *MAE* produced by the ANN model is considered, and values that are close to 0 suggest a successful forecast. Meanwhile, *RMSE* stands for residual mean square error, and it can be used to measure how well the model predicts the response as well as how widely distributed the residuals are. A better match is indicated by lower *RMSE* values. Additionally, coefficient of determination (R^2^) was employed to determine whether differences in one variable can be explained by variations in another variable. 

## 3. Results and Discussion

### 3.1. Exploratory Analysis by Sites

High variability among the nine investigated vineyards for pomological traits (i.e., berry weight (W), berry length (L), berry diameter (D), berry adherence strength, berry length/berry diameter, and berry CIELab color coordinates), minimum, maximum, and means values were observed (Table 1) as well as coefficient of variation, skewness, and Kurtz. In detail, the berry weight values varied from 3.20 to 3.66 g in vineyards V1 and V9, respectively, as shown in Table 2, with a mean value of 3.46 ± 0.16 g. This suggested that no large disparity was seen in berry weight as the CV value was 4.59%, as shown in Table 1. Previous results showed that various agronomical management and other practices can lead to an increase in berry weight [41,42,43,44,45]. In addition, Caspari et al. [46] reported that the removal of leaves increased the berry weight of ‘Sauvignon blanc’ grapes. Vineyards V1 and V9 denote the lowest and greatest values of berry length and berry diameter (L: 1.9 and 2.8 cm; D: 2.0 and 3.0 cm, respectively). The higher the berry growth rate, early uptake of glucose, sucrose, and fructose, and increase in absolute berry water content, then the larger berry length and berry diameter [47].

Further, the berry length values varied from 1.9 to 4.4 cm in vineyards V1 and V9, respectively, as shown in Table 2, with a mean value of 2.4 ± 0.29 cm (Table 1). This suggested that no large variation was seen in berry length as the CV value was 12.07%, as shown in Table 1. Additionally, the berry diameter values varied from 2.0 to 3.0 cm in vineyards V1 and V9, respectively, as shown in Table 2, with a mean value of 2.5 ± 0.32 cm (Table 1). This suggested that no large disparity was seen in berry diameter as the CV value was 12.42%, as shown in Table 1. Furthermore, the shape index of berries (berry length/berry diameter) was obtained from the L and D ratio and ranged from approximately 0.90 to 0.96, as shown in Table 2 (vineyards V5 and V6, respectively), with a mean value of 0.93 ± 0.01 (Table 1). This suggested that no large disparity was seen in the shape index of berries as the CV value was 1.55%, as shown in Table 1. Moreover, berry adherence strength showed reasonable variability among the vineyards. In detail, the berry adherence strength values varied from 4.22 to 6.33 N, as shown in Table 1, with a mean value of 5.22 ± 0.68 N cm (Table 1). The highest and the lowest berry adherence strengths were seen for berries cultivated in vineyards V1 and V9, respectively (Table 2). It is obvious that from Table 2, the adherence strength of Flame Seedless’ connection was altered depending on the vineyard, with large variations between them. It might be argued that berries’ brush and pedicel have more persistent insoluble pectin content, which accounts for their increased adherence strength [48]. Berry adherence strength and firmness changes are tightly correlated with parameters affecting weight and water loss [49]. However, the cation Ca^2+^ encourages the firmness of the cell wall by chelating the free carboxylic groups of galacturic units and cross-linking the pectic polysaccharide chains, forming a firmer, tighter structure [47]. Similar physical trait variability was reported for Flame Seedless grapes by other authors [50,51,52,53,54]; however, a comparison of the supplied results with the literature data was not possible.

Fruit color coordinates showed high variability among the vineyards; L* ranged from 25.65 (vineyard V9) to 46.45 (vineyard V1), as shown in Table 2, with a mean value of 35.13 ± 6.79 (Table 1). This suggests that a large disparity was seen for the L* of berries, as the CV value was 19.3%, as shown in Table 1. Additionally, a* ranged from 5.24 (vineyard V1) to 10.99 (vineyard V9), as shown in Table 2, with a mean value of 8.58 ± 1.72 (Table 1). This suggests that a large disparity was seen for the a* of berries, as the CV value was 20.10%, as shown in Table 1. Furthermore, b* ranged from 3.12 (vineyard V9) to 12.96 (vineyard V1), as shown in Table 2, with a mean value of 7.83 ± 3.18 (Table 1). This suggests that a large disparity was seen for the b* of berries, as the CV value was 40.61%, as shown in Table 1. The high variability among the nine investigated vineyards for CIELab color coordinates, shown in Table 1, is cleared based on coefficients of variation values, which were 19.33%, 20.10%, and 40.61% for L*, a*, and b*, respectively. However, there are agricultural practices, such as stimulant fertilizers, which have recently gained popularity due to their enhancement of berry coloration, for example, potassium sources [55], kaolin foliar fertilizer [56], and algal extract [57]. The color of the berries of vines cultivated in V9 was visually more red (a* = 10.88) than in other vineyards at harvest time. It could be suggested that a* correlated with anthocyanin content. However, anthocyanin is reflected in the berry’s skin color in Crimson Seedless grapes. It accumulates in berries at the beginning of the véraison stage of berry development. Continually, the accumulation during berry development is related to abscisic acid (ABA) metabolism, by which berry skin anthocyanin content increases and color appears [58]. Further, by application of exogenous cyanocobalamin, the skin color profile of Crimson Seedless grapevines can be improved [59]. Furthermore, it may be that Vitamin B_12_ activated cDNA and soluble protein in plant cells during berry development [60], which then increased the accumulation of peonidin and the acylated derivatives of anthocyanin content in fresh grape skins [61].

**Figure 3:**
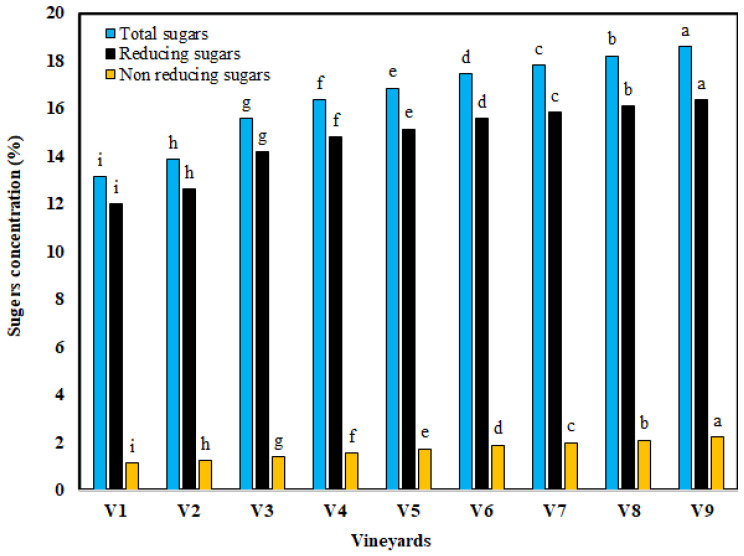
Distribution of mean values of total sugars, reducing sugars, and non-reducing sugars of Flame Seedless grape berries cultivated under various agronomical management and other practices in investigated vineyards. (Different letters indicate that the means are significantly different from each other (*p* < 0.05)).

Variability among the nine vineyards for chemical parameters’ (i.e., TSS, titratable acidity, TSS/titratable acidity, anthocyanin, total sugars, reducing sugars, and non-reducing sugar) were observed (Table 1), which indicates minimum, maximum, and means values as well as the coefficient of variation, skewness, and Kurtz. The distribution of mean values of such attributes for the different vineyards are shown in Table 3 and in Figure 3. As regards the TSS, the values ranged from 16.70% to 22.10%, as shown in Table 3, with a mean value of 19.48 ± 1.49% (Table 1). This suggested that no variation was seen for the TSS of berries, as the CV value was 9.94%, as shown in Table 1. The highest and the lowest TSS were seen for berries cultivated in vineyards V1 and V9, respectively (Table 3). When high-potassium fertilization was applied, the TSS of the Crimson Seedless grape increased [44]. Moreover, Bledsoe et al. [62] found that the TSS in the Sauvignon blanc variety was significantly higher in berries from vines with leaves removed.

Vineyard V1 presented the highest mean titratable acidity value (0.78%), whereas vineyard V9 presented the lowest one (0.48%), as shown in Table 3. Moreover, the range was 0.48% to 0.70%, with a mean value of 0.62 ± 0.09% (Table 1). This suggested that high variation was seen for the titratable acidity of berries, as the CV value was 15.30%, as shown in Table 1. The decrease in the titratable acidity of juice caused by potassium foliar application as the potassium source improves the sugar transport into berries [63]. Additionally, Ethephon’s capacity to increase membrane permeability and speed up the respiration of acids stored in cell vacuoles may be the cause of the acidity reduction that results from its application [47]. Similar trends for TSS and titratable acidity were reported in [47,63]. The fluctuation in titratable acidity and TSS due to various environmental conditions and site-specific field management strategies (plant variety, planting date, harvest time, and cultural technique) was also reported by Cocco et al. [64].

The study of the TSS/titratable acidity ratio is regarded as a trustworthy indicator of grape maturity, and a significant rise indicates the onset of ripening [47]; also, it is a better index for fruit customer acceptance, as reported by Crisosto et al. [65]. However, the concentration of titratable acidity, TSS, and their ratios are not stationary but fluctuate considerably during fruit maturation and ripening. Because of this, these features are frequently utilized as commercial and laboratory indicators of maturity for numerous horticultural crops [66,67]. In this study, the TSS/titratable acidity values ranged from 21.14 to 46.04%, as shown in Table 1, with a mean value of 32.62 ± 8.08 (Table 1). This suggested that high disparity was seen for the TSS/titratable acidity of berries, as the CV value was 24.78%, as shown in Table 1. The highest and the lowest TSS/titratable acidity were seen for berries cultivated in vineyards V9 and V1, respectively (Table 3).

High variability in anthocyanin was observed (Table 1), as denoted by the coefficient of variation of 23.95%. Vineyards V1 and V9 showed the minimum and maximum values, respectively (17.34 and 38.81 mg/100 g fruit weight), as shown in Table 3. However, the mean value was 28.58 ± 6.84 mg/100 g of fruit weight (Table 1). During development, the skin’s anthocyanin content rises, enhancing the berry’s color [68]. The reduction in nitrogen dose, foliar potassium, foliar ethereal, and basal leaf removal may be the causes of the improvement in anthocyanin [63]. Following foliar treatments of phosphor and potassium, Topalović et al. [69] noted an increase in total anthocyanins. Additionally, Abd El-Razek et al. [44] claimed that fertilizing Crimson Seedless grapes with a high dose of potassium and low nitrogen levels boosted anthocyanin levels. When grapevine berries ripen, ethylene levels rise right before veraison, encouraging some of the linked processes, such as anthocyanin accumulation, according to Dal Ri et al. [70]. Monitoring anthocyanin concentrations in connection to various agronomical management is crucial for assessing the quality of all fruits since, if present in high concentrations, anthocyanins may contribute to the red color of fruits as the plant pigments. The striking red color of table grapes is a result of anthocyanins [71]. Additionally, the controlling of anthocyanin concentration in crop loads resulted in the proper coloration of ‘Aki Queen’ fruits through the regulation of anthocyanin concentration in grapes [72].

As regards total sugars, it showed moderate disparity (Table 1), as denoted by the CV of 11.04%, the maximum total sugars of 18.59% was revealed in vineyard V9, whereas the minimum total sugars was revealed in vineyard V1 (13.14%), as shown in Figure 3. However, the mean value of total sugars was 16.43 ± 1.81% (Table 1). Additionally, as regards reducing sugars, a small variation was observed (Table 1), as denoted by the CV of 9.89%, however, the maximum reducing sugars of 16.52%, the minimum reducing sugars of 11.96, and as shown in Figure 3; the maximum and the minimum reducing sugars were noticed in vineyard V9 and V1, respectively. Furthermore, as regards non-reducing sugars, high variation was observed (Table 1), as denoted by the CV of 21.82%. However, non-reducing values were in the range of 1.12% to 2.24%, with a mean value of 1.69 ± 0.37% (Table 1); the maximum and the minimum non-reducing sugars were noticed in vineyard V9 and V1, respectively (Figure 3). Similar to total sugars, the reducing sugars and non-reducing sugars’ variability was reported for Flame Seedless grapes by another author [2], but a comparison of the presented data with the literature data was not possible. The nine investigated vineyards showed an identical trend for the total sugars, reducing sugars, and non-reducing sugars; however, the highest values of them belonged to vineyard V9 and the lowest values belonged to vineyard V1. King et al. [73] observed that Hawke’s Bay grapes had more sugar concentration after crop removal. Somkuwar et al. [74] noticed that when the number of clusters per vine in grape cv. Jumbo Seedless increased, the concentration of reducing sugars decreased. Additionally, reducing, non-reducing, and total sugars concentrations of grape berries increased with increasing concentrations of micronutrients by applying agronomical management, such as fertilization, additionally, the increase in the sugar content of grape berries could be associated with the increased chlorophyll content [2].

### 3.2. Correlation Analysis

One of the most crucial competitive market factors is fruit quality, which has an impact on both the price and volume of fruit sold [75]. Multiple variables contribute to its occurrence, including the individual and cumulative effects of mineral nutrients [76]. The link or association between fruit quality characteristics was examined using Pearson’s correlation coefficient test (Table 4). With varying r-values, all fruit quality criteria were connected with one another either positively or negatively; r-values were in the high positive range of 0.921 to 0.995. As an example, high positive correlations between berry weight and berry length, berry diameter, berry a*, TSS, TSS/titratable acidity, anthocyanin, total sugars, reducing sugars, and non-reducing sugars, were observed by r-values of 0.988, 0.989, 0.986, 0.985, 0.976, 0.992, 0.983, 0.972, and 0.995, respectively. This suggests that increasing berry weight increases the berry length, berry diameter, berry a*, TSS, TSS/titratable acidity, anthocyanin, total sugars, reducing sugars, and non-reducing sugars. In another study, Pilar et al. [77] found a positive correlation between TSS and 100 berry weight. The high negative correlation between berry weight and berry L*, berry b*, berry adherence strength, and titratable acidity were noticed by r-values of −0.993, −0.991, −0.990, and −0.989, respectively. Furthermore, anthocyanin presented a negative correlation with L*, and this agreed with the results of Peppi et al. [78] and Mekawy and Ahmed [79]. Given that anthocyanin and color attributes have a link, it can be assumed that colored grapes have higher anthocyanin concentrations [79]. Additionally, total sugars and TSS have a high positive correlation, seen by the r-value of 0.948, also, this finding was seen by Mekawy and Ahmed [79].

### 3.3. Prediction of Flame Seedless Quality Attributes

For the purpose of identifying the ideal network architecture, different ANN configurations were created and contrasted with one another (input-hidden-output layers). Three input data were present in the network’s first layer, and its only output layer represented the quality attributes. In fact, how well a dataset may be learned may depend on how many hidden neurons there are [80]. The network will not be able to learn if there are not enough neurons used. Overfitting can occur when there are too many hidden neurons, which prevents the generalizability of the input/output relationship but improves network learning and data memorization [80,81]. An increase in hidden neurons may enhance ANN performance [81].

We examined various neurons in the ANN’s hidden layer (from 1 to 30) to determine the best topology for the ANN, and we chose the topology with the highest overall accuracy (Figure 2) based on the smallest RMS Errors and the highest correlation coefficients of training and testing sets. The best model was built using the sigmoid activation function for hidden neurons and 20 neurons in the hidden layer. For each quality parameter prediction of Flame Seedless grapes berries utilizing ANN models by 3-20-11 topology and testing set, Table 5 displays the errors analysis and coefficients of determination (R^2^), however R^2^ determines whether differences in one variable can be explained by variations in another variable. 

**Figure 4:**
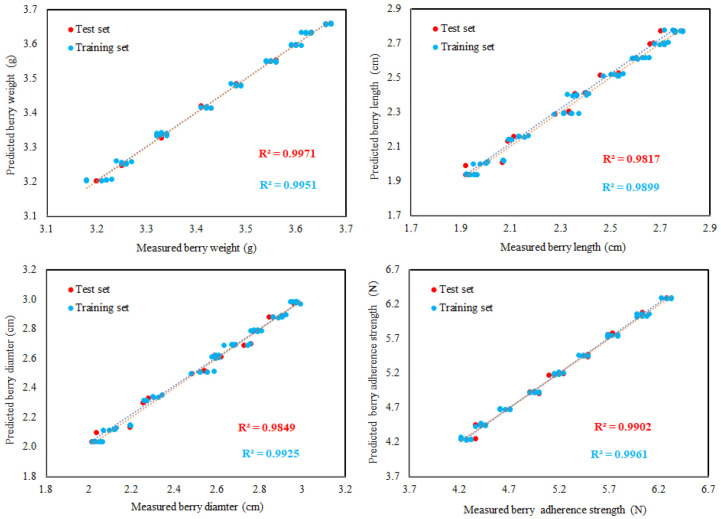
Predicted vs. experimental values of berry weight, berry length, and berry diameter berry adherence strength using the optimal ANN topology and training and testing sets. The coefficients of determination (R^2^) are reported.

**Figure 5:**
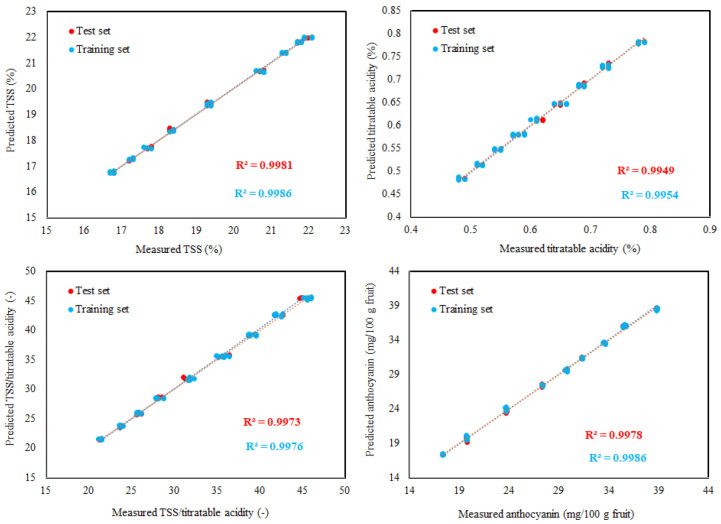
Predicted vs. experimental values of the TSS, titratable acidity TSS/acidity, and anthocyanin using the optimal ANN topology and training and testing sets. The coefficients of determination (R^2^) are reported.

**Figure 6:**
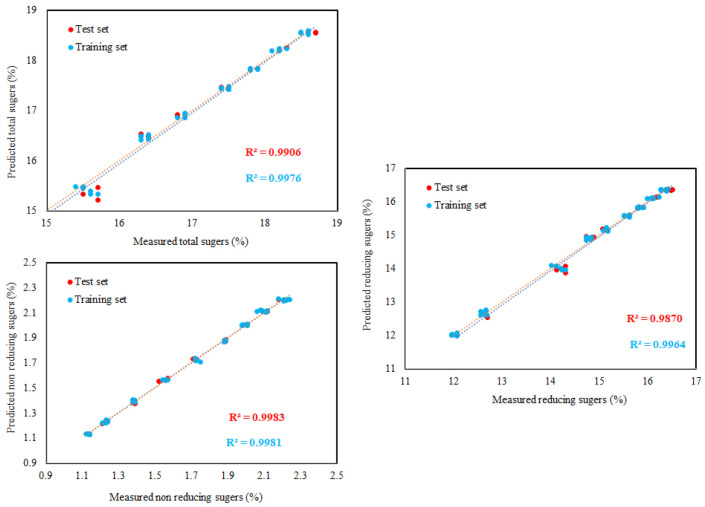
Predicted vs. experimental values of the total sugars, reducing sugars, and non-reducing sugars using the optimal ANN topology and training and testing sets. The coefficients of determination (R^2^) are reported.

Using the ideal ANN topology, there was generally good agreement between the experimental and predicted values as shown by higher values of R^2^ (Table 5). Meanwhile, to appraise the ability of the established ANN model well, we compare the predicted and the experimental values of all quality attributes in the training and testing phases (Figure 4, Figure 5 and Figure 6). The results display that the distribution shape of the predicted quality attributes’ values is very close to the experimental quality attributes’ values in scatter plots (Figure 4, Figure 5 and Figure 6), and all predicted and measured quality attributes’ values have a similar trend. The results indicate that the established ANN model is consistent and effective for predicting the quality attributes of Flame Seedless grapes berries. In reality, as illustrated in Figure 4, Figure 5 and Figure 6, the R^2^ using the test dataset and training dataset were all above 0.90. This result shows that the established ANN model was adequate for solving the nonlinearity of the relationships among CIELab colorimeter parameters of berries L*, a*, and b* and Flame Seedless grapes berries’ quality attributes (berry weight, berry length, and berry diameter as well as berry adherence strength, anthocyanin, TSS, TSS/titratable acidity, total sugars, titratable acidity, reducing sugars, and non-reducing sugars). In particular, the low values of *MAE* and *RMSE* for the testing set (Table 5) indicated a high capacity for predicting the quality attributes of Flame Seedless grape berries due to a very small dispersion of residuals. ANNs are useful tools for assisting customers in selecting the appropriate fruits for various uses, as well as for improving postharvest life, final quality, and maintaining fruit quality for better marketability in the fruit field, such as sorting, classification, grading, identification, and quality feature prediction. In the studies by Amoriello et al. [28] and Yoshioka et al. [82], they discovered that ANN models could accurately predict anthocyanins by taking into account the CIELab coordinates of L*, a*, and b*. A recent study suggested that ANN modeling can be successfully exploited for the prediction of quality parameters of winter rapeseed [83]. Furthermore, ANN was developed to predict the TSS, titratable acidity, TSS/titratable acidity, anthocyanin, vitamin C, and total carotenoids contents using surface-color CIELab coordinates of L*, hue, and chroma for fresh peach fruit based on inputs of juice volume, single fruit weight, and sphericity percent [84]. In addition, ANN could be employed as a tool for the identification of peach varieties based on physical characteristics [85]. Finally, ANN can be utilized as an alternative tool for fruit mass prediction of ber fruits (*Ziziphus mauritiana* Lamk) [86] and peach fruits [87].

### 3.4. Relevance of the Input Variables to the Predictions Made by the ANN Model

To determine how important the input variables are, the relative contribution of the input variables to the predictions made by the ANN model was evaluated. using contribution analysis by Qnet2000 software. The relative importance (%) of the CIELab coordinates for the network and each quality metric was then ranked (Table 6 and Table 7). In the ANN model, higher significance values indicate that the input variable is given more weight. As shown in Table 6 and Table 7, berry b* was the most significant variable for berry weight, berry adherence strength, TSS, TSS/titratable acidity, titratable acidity, total sugars, reducing sugars, and non-reducing sugars; a* for anthocyanin, berry length, and berry diameter. The fruit components’ chemical makeup may help to explain it. Fruit pigment buildup and variations in the amount of sugar and organic acid cause a correlation between color and fruit quality [88,89]. A number of biochemical and physiological processes that take place during fruit ripening also cause color changes depending on the cultivar [90,91]. The color of the skin and pulp is specifically caused by various bioactive substances, such as anthocyanins, carotenoids, and polyphenols. For instance, anthocyanins and polyphenols are primarily associated with the hues of purple and red [89].

Berry skin color is a significant quality parameter in table grapes. However, weak berry color expansion in red cultivars, such as Flame Seedless, Red Globe, and Crimson Seedless, leads to poor quality and serious economic loss [63]. The unique buildup of anthocyanins during the ripening of these grapes is what gives them their signature red hue. The study’s use of ANN predictions was shown to be much more reliable when a large number of data points were used for ANN modeling. The proper training and testing of ANNs require a wide variety of data [81]. The values of the several parameters in our analysis varied widely and were spread across all intervals rather evenly. However, utilizing a small sample size may have indicated a weakness in the estimation of the Flame Seedless grape berries’ quality characteristics, which could be one of the reasons why certain models produced inaccurate results [92].

## 4. Conclusions

In this study, we use the feed-forward architecture as a multi-layered perceptron, with back propagation as a training algorithm to build an effective and reliable artificial neural network model that can predict the quality attributes of Flame Seedless grapes cultivated under various agronomical management and other practices by the CIELab colorimeter parameters of berries L*, a*, and b*. The established ANN model had the best topology of 3-20-11 as it reaches the highest accuracy (R^2^ above 0.9 for all investigated quality parameters). High positive correlation between berry weight and berry length, berry diameter, berry a*, TSS, TSS/titratable acidity, anthocyanin, total sugars, reducing sugars, and non-reducing sugars, were observed by r-values of 0.988, 0.989, 0.986, 0.985, 0.976, 0.992, 0.983, 0.972, and 0.995, respectively. Furthermore, the anthocyanin presented a negative correlation with L*. We conducted a relative contribution analysis by Qnet2000 software, and the results display that the content of the berry b* was the most significant variable by relative importance percentage for berry weight (68.95%), berry adherence strength (49.65%), TSS (73.48%), TSS/titratable acidity (57.44%), titratable acidity (46.6%), total sugars (62.9%), reducing sugars (64.86%), and non-reducing sugars (50.98%). Meanwhile, a* was the most significant variable as the relative importance percentage for anthocyanin (37.47%), berry length (45.28%), and berry diameter (47.65%). According to the study’s findings, colorimetric measurements are a promising nondestructive, quick, and affordable tool for the quick assessment of Flame Seedless grape berries’ quality. As a result, grape growers and technicians in the food processing industry can successfully use it with an ANN model in commercial applications.

## Figures and Tables

**Table 1:** Statistical criteria of experimental results of quality attributes of Flame Seedless grape berries cultivated under various agronomical management and other practices.

Quality Attributes	Statistical Criteria						
	Minimum	Maximum	Mean	Standard Deviation	Coefficient of Variation, CV (%)	Skewness	Kurtz
Berry weight (g)	3.18	3.67	3.46	0.16	4.59	−0.31	−1.33
Berry length (cm)	1.9	2.8	2.4	0.29	12.07	−0.19	−1.34
Berry diameter (cm)	2.0	3.0	2.5	0.32	12.42	−0.35	−1.20
Berry length/berry diameter	0.90	0.96	0.93	0.01	1.55	−0.25	−0.41
Berry L*	25.65	46.45	35.13	6.79	19.33	0.18	−1.35
Berry a*	5.24	10.99	8.58	1.72	20.10	−0.48	−0.80
Berry b*	3.12	12.96	7.83	3.18	40.61	0.07	−1.28
Berry adherence strength (N)	4.22	6.33	5.22	0.68	12.96	0.12	−1.28
TSS (%)	16.70	22.10	19.48	1.94	9.94	−0.02	−1.63
Titratable acidity (%)	0.48	0.79	0.62	0.09	15.30	0.23	−1.07
TSS/titratable acidity (ࢤ)	21.14	46.04	32.62	8.08	24.78	0.19	−1.33
Anthocyanin (mg/100 g fresh fruit)	17.30	38.90	28.58	6.84	23.95	−0.24	−1.10
Total sugars (%)	13.10	18.70	16.43	1.81	11.04	−0.65	−0.89
Reducing sugars (%)	11.96	16.52	14.74	1.46	9.89	−0.78	−0.73
Non-reducing sugars (%)	1.12	2.24	1.69	0.37	21.82	−0.13	−1.38
No. of points	81	81	81	81	81	81	81

**Table 2:** Distribution of mean values of weight, length, diameter, adherence strength, and color space coordinates (L*, a*, and b*) of Flame Seedless grape berries cultivated under various agronomical management and other practices.

Vineyards	Berry Weight	Berry Length	Berry Diameter	Berry Adherence Strength	Berry Length/Berry Diameter (Shape Index)	Berry L*	Berry a*	Berry b*
	(g)	(cm)	(cm)	(N)	(−)	(−)	(−)	(−)
V1	3.20 ^i^	1.9 ^i^	2.0 ^i^	6.29 ^a^	0.950 ^a^	45.90 ^a^	5.32 ^i^	12.83 ^a^
V2	3.26 ^h^	2.0 ^h^	2.1 ^h^	6.03 ^b^	0.940 ^b^	43.16 ^b^	6.74 ^h^	11.51 ^b^
V3	3.33 ^g^	2.1 ^g^	2.3 ^g^	5.72 ^c^	0.930 ^c^	40.76 ^c^	7.48 ^g^	10.18 ^c^
V4	3.42 ^f^	2.3 ^f^	2.5 ^f^	5.46 ^d^	0.920 ^de^	37.88 ^d^	8.24 ^f^	9.07 ^d^
V5	3.48 ^e^	2.4 ^e^	2.6 ^e^	5.18 ^e^	0.916 ^e^	33.83 ^e^	8.86 ^e^	7.92 ^e^
V6	3.55 ^d^	2.5 ^d^	2.7 ^d^	4.96 ^f^	0.928 ^cd^	31.88 ^f^	9.39 ^d^	6.39 ^f^
V7	3.60 ^c^	2.6 ^c^	2.8 ^c^	4.65 ^g^	0.937 ^bc^	29.37 ^g^	9.91 ^c^	5.22 ^g^
V8	3.62 ^b^	2.7 ^b^	2.9	4.41 ^h^	0.930 ^c^	27.60 ^h^	10.38 ^b^	4.12 ^h^
V9	3.66 ^a^	2.8 ^a^	3.0 ^ab^	4.28 ^i^	0.927 ^cd^	25.77 ^i^	10.88 ^a^	3.19 ^i^
LSD (5%)	0.0088	0.0292	0.0307	0.0377	0.0091	0.271	0.084	0.052

Different letters indicate that means are significantly different from each other (*p* < 0.05).

**Table 3:** Distribution of mean values of internal quality (TSS, titratable acidity, TSS/acidity, and anthocyanin) of Flame Seedless grape berries cultivated under various agronomical management and other practices.

Vineyards	TSS	Titratable Acidity	TSS/Titratable Acidity	Anthocyanin
	%	%	(ࢤ)	(mg/100 g Fruit Weight)
V1	16.76 ^i^	0.78 ^a^	21.39 ^i^	17.34 ^i^
V2	17.26 ^h^	0.73 ^b^	23.78 ^h^	19.76 ^h^
V3	17.72 ^g^	0.68 ^c^	25.89 ^g^	23.72 ^g^
V4	18.36 ^f^	0.65 ^d^	28.24 ^f^	27.36 ^f^
V5	19.36 ^e^	0.61 ^e^	31.68 ^e^	29.73 ^e^
V6	20.73 ^d^	0.58 ^f^	35.75 ^d^	31.36 ^d^
V7	21.36 ^c^	0.55 ^g^	39.15 ^c^	33.58 ^c^
V8	21.74 ^b^	0.52 ^h^	42.18 ^b^	35.53 ^b^
V9	22.00 ^a^	0.48 ^i^	45.52 ^a^	38.81 ^a^
LSD (5%)	0.0588	0.0057	0.3569	0.0725

Different letters indicate that means are significantly different from each other (*p* < 0.05).

**Table 4:** Pearson’s correlation coefficients (r) describing the correlations among quality variables of Flame Seedless grape cultivated under various agronomical management and other practices.

Parameters	Berry Weight	Berry Length	Berry Diameter	L*	a*	b*	Berry Adherence Strength	TSS	Titratable Acidity	TSS/Titratable Acidity	Anthocyanin	Total Sugars	Reducing Sugars
Berry weight	1												
Berry length	0.988	1											
Berry diameter	0.989	0.993	1										
L*	−0.993	−0.990	−0.987	1									
a*	0.986	0.980	0.986	−0.987	1								
b*	−0.991	−0.988	−0.982	0.995	−0.986	1							
Berry adherence strength	−0.990	−0.988	−0.984	0.995	−0.986	0.997	1						
TSS	0.985	0.980	0.966	−0.988	0.965	−0.989	−0.985	1					
Titratable acidity	−0.989	−0.985	−0.985	0.993	−0.994	0.995	0.993	−0.977	1				
TSS/titratable acidity	0.976	0.978	0.966	−0.985	0.965	−0.993	−0.988	0.988	−0.985	1			
Anthocyanin	0.992	0.987	0.992	−0.992	0.991	−0.992	−0.992	0.971	−0.994	0.978	1		
Total sugars	0.983	0.970	0.983	−0.974	0.986	−0.971	−0.973	0.948	−0.978	0.940	0.985	1	
Reducing sugars	0.972	0.957	0.973	−0.960	0.978	−0.956	−0.959	0.930	−0.967	0.921	0.975	0.998	1
Non-reducing sugars	0.995	0.992	0.988	−0.997	0.983	−0.996	−0.995	0.990	−0.992	0.989	0.993	0.974	0.959

**Table 5:** RMSE and MAE values and R^2^ for each quality parameters predictions of Flame Seedless grapes berries using ANN model with a 3-20-11 topology and testing set.

Quality Parameters	*RMSE*	*MAE*	R^2^
Berry weight (g)	0.008	0.007	0.9971
Berry length (cm)	0.042	0.036	0.9817
Berry diameter (cm)	0.038	0.033	0.9849
Berry adherence strength (N)	0.065	0.058	0.9902
TSS (%)	0.083	0.069	0.9981
Titratable acidity (%)	0.007	0.006	0.9949
TSS/titratable acidity (ࢤ)	0.453	0.371	0.9973
Anthocyanin (mg/100 g fruit weight)	0.313	0.248	0.9978
Total sugars (%)	0.172	0.134	0.9906
Reducing sugars (%)	0.162	0.127	0.9870
Non-reducing sugars (%)	0.015	0.011	0.9983

**Table 6:** Relative importance (%) of the input variables for ANN model predictions of berry weight, berry length, berry diameter, berry adherence strength, and TSS.

Input Variables	Output Variables				
	Berry Weight	Berry Length	Berry Diameter	Berry Adherence Strength	TSS
Berry L*	4.24	9.78	26.73	11.78	7.29
Berry a*	26.81	45.28	47.65	38.57	19.23
Berry b*	68.95	44.93	25.61	49.65	73.48

**Table 7:** Relative importance (%) of the input variables for ANN model predictions of titratable acidity, TSS/titratable acidity, anthocyanin, total sugars, reducing sugars, and non-reducing sugars.

Input Variables	Output Variable					
	Titratable Acidity	TSS/Titratable Acidity	Anthocyanin	Total Sugars	Reducing Sugars	Non-Reducing Sugars
Berry L*	10.17	11.21	28.47	9.62	13.24	19.27
Berry a*	43.21	31.34	37.47	27.48	21.9	29.75
Berry b*	46.63	57.44	34.06	62.9	64.86	50.98

## Data Availability

All data are presented in this article in the form of figures and tables.
